# Vascular Complications of Systemic Sclerosis during Pregnancy

**DOI:** 10.1155/2010/287248

**Published:** 2010-08-11

**Authors:** Eliza F. Chakravarty

**Affiliations:** Division of Immunology and Rheumatology, Stanford University School of Medicine, 1000 Welch Road, Suite 203, Palo Alto, CA 94304, USA

## Abstract

Systemic sclerosis (SSc) is a chronic autoimmune disorder characterized by progressive fibrosis of the skin and visceral tissues as well as a noninflammatory vasculopathy. Vascular disease in systemic sclerosis is a major cause of morbidity and mortality among nonpregnant patients with SSc and is even a bigger concern in the pregnant SSc patient, as the underlying vasculopathy may prevent the required hemodynamic changes necessary to support a growing pregnancy. Vascular manifestations including scleroderma renal crisis and pulmonary arterial hypertension should be considered relative contraindications against pregnancy due to the high associations of both maternal and fetal morbidity and mortality. In contrast, Raynaud's phenomenon may actually improve somewhat during pregnancy. Women with SSc who are considering a pregnancy or discover they are pregnant require evaluation for the presence and extent of underlying vasculopathy. In the absence of significant visceral vasculopathy, most women with SSc can expect to have reasonable pregnancy outcomes.

## 1. Introduction

Systemic sclerosis (SSc) is a chronic autoimmune disorder characterized by progressive fibrosis of the skin and visceral tissues as well as a non-inflammatory vasculopathy manifesting as Raynaud's phenomenon, digital ulcerations, pulmonary arterial hypertension, and scleroderma renal crisis. Vascular disease in systemic sclerosis is a major cause of morbidity and mortality among non-pregnant patients with SSc. There are two main subsets of SSc: diffuse cutaneous disease is characterized by cutaneous fibrosis proximal to the forearms and is associated with higher incidence of scleroderma renal crisis and pulmonary fibrosis; limited cutaneous disease has more limited cutaneous involvement to the distal extremities and face and is associated with higher prevalence of digital ulcers and pulmonary arterial hypertension. 

Akin to many autoimmune diseases, SSc has a strong female predominance, with an approximate female- to male- ratio of 5 : 1 [1, 2]. SSc is a relatively rare disease, with a prevalence in North America estimated to be approximately 49,000–55,000, between 25 and 44 per 100,000 persons [1, 2]. Symptom onset of SSc usually begins in the 5th decade, approximately 10 years prior to the mean age of menopause. In more recent decades, many women have postponed childbearing into their 30 s and 40 s for career and other personal reasons. For this reason, the number of women who develop SSc and have not yet completed their families and may be considering pregnancy is likely to increase. When counseling women with SSc who are considering pregnancy or who discover a pregnancy, it is critical to understand the potential vascular complications that may lead to adverse pregnancy outcomes.

## 2. Normal Hemodynamic and Vascular Changes of Pregnancy

In order to accommodate the enlarging uterus and growing fetus, a pregnant woman's body must make numerous physiologic hemodynamic changes. Many of these changes involve changes in blood volume, vascular resistance, cardiac output, and oxygen consumption. Most physiologic changes begin early in gestation and peak by the late second trimester of pregnancy. Although not definitive, it has been suggested that an initial fall in systemic vascular resistance leads to the other well-established vascular and hemodynamic changes of pregnancies [3]. Activation of the renin-angiotensin-aldosterone system results in a 7-8 liter increase in total body fluid during gestation (distributed between maternal intra- and extracellular spaces, fetus, and amniotic fluid) [4]. Cardiac output increases by 50% over the course of pregnancy, with the majority of increase occurring within the first 8 weeks, followed by a gradual increase until plateau mid-third trimester [5]. This is mediated by an increase in both stroke volume early in pregnancy and heart rate throughout pregnancy. During pregnancy, cardiac output is preferentially distributed to the uterus, kidneys, skin, and breast tissue and away from skeletal muscle [6]. The pulmonary system additionally undergoes significant physiologic changes during pregnancy. Pulmonary vascular resistance decreases. Basal oxygen consumption increases by 50 mL/min by term, and alveolar ventilation increases, lowering PCO2 by about 8 torr.

The exact mechanisms underlying the hemodynamic changes during pregnancy remain to be completely understood. An increase in prostacyclin in both the fetoplacental tissues and maternal system plays a clear role in systemic vasodilation; however, additional mediators are required to explain the degree of reduction in vascular resistance [3]. Nitric oxide (NO) is likely candidate to play a significant role in the hemodynamic accommodations during pregnancy. There appears to be a permanent basal activity of NO in utero- and fetoplacental vasculature in pregnant women; the trophoblast may be a significant source of NO production [3]. NO donors administered to women between 8 and 10 weeks gestation resulted in a further fall of peripheral resistance [7]. Other studies have demonstrated a deficiency of NO in pregnancy complications associated with defective placental perfusion: preeclampsia and intrauterine growth restriction [3]. In addition to the important roles of prostacyclin and NO in the maintenance of low peripheral vascular resistance, the maternal vasculature appears to be resistant to the effects of angiotensin II.

Physiologic hemodynamic changes are an integral part of human pregnancy. In some cases, changes in vascular blood flow and decreased vascular resistance may lead to improvement in manifestations of systemic sclerosis. In other cases, an intrinsic inability to adapt to the necessary hemodynamic changes in pregnancy, possibly due to underlying vasculopathy of systemic sclerosis, may contribute to adverse pregnancy outcomes including hypertensive disorders of pregnancy and intrauterine growth restriction.

## 3. Maternal and Fetal Outcomes of the SSc Pregnancy

Given the prominent vasculopathy associated with SSc, there is concern for an increased risk of adverse pregnancy outcomes associated with vascular compromise or inability to make appropriate hemodynamic adaptations to support a pregnancy. Indeed, early reports suggested very high rates of maternal death during pregnancy [8–11]. It is likely for this reason that women with SSc in past decades had been strongly advised against pregnancy and often counseled to terminate pregnancies that have occurred [12]. More recently, a series of retrospective and prospective studies have provided more detailed analysis of pregnancy outcomes and have demonstrated that, for most women with SSc, pregnancy outcomes are reasonably good [13–18]. Reported rates of early pregnancy loss of 14%*‒*15% [13, 14] are somewhat increased from the estimated 10% in the general population. Late pregnancy losses were few, and generally occurred in women with severe diffuse SSc [14, 16]. Preterm delivery rates have ranged from 8%to40% [13, 14, 16]. The majority of preterm deliveries were on or after gestational age 34 [14]. Small for gestational age infants (<10th% tile for gestational age) [19], ranged from 0% to 50% [2, 14, 16]. Cases of preeclampsia were isolated [14, 16]. It must be remembered, however, that these studies reflect the experience of approximately 200 patients followed at tertiary-care centers over a period of 10 years. A population-based study of pregnancy outcomes among women with SSc in the United States that utilized administrative hospital discharge databases identified 504 SSc women who delivered between 2004 and 2006 [18]. This study found a 22.9% rate of hypertensive disorders including preeclampsia, a four-fold increased odds compared to the general population (85% CI, 2.4–6.6). Similarly, a nearly four-fold increased rate of intrauterine growth restriction was found. Overall, the majority of patients with SSc appear to have reasonable obstetric outcomes although women with rapidly progressive diffuse disease may be at higher risk for complications.

## 4. Scleroderma Renal Crisis

Scleroderma renal crisis, characterized by malignant hypertension, proteinuria, acute renal failure, microangiopathic changes, and the pathognomonic “onion skin” appearance of renal arteries on pathology, is one of the most severe complications of SSc [20]. Affecting 5%–10% of SSc patients, renal crisis characteristically occurs in patients with rapidly progressing diffuse skin disease of relatively recent onset. Prior to the widespread use of angiotensin-converting enzyme (ACE) inhibitors, renal crisis was one of the leading causes of mortality. Many of the perinatal deaths reported among SSc patients involved scleroderma renal crisis [7–10]. Even in more recent series, episodes of renal crisis during pregnancy were reported. Steen et al. reported two cases of scleroderma renal crisis in a retrospective study of 86 pregnancies occurring after the diagnosis of SSc [12]. Both cases occurred abruptly in the third trimester of pregnancy and resulted in preterm delivery. One woman developed end- stage renal disease, and the other died from status epilepticus. In a prospective study of 91 pregnancies, Steen reported two additional cases of renal crisis [13]. Both women required hemodialysis after delivery. All of these cases occurred in women with early, rapidly progressive diffuse disease. It remains unclear if rates of renal crisis are increased in pregnant women compared to nonpregnant women with severe diffuse disease. The severity of scleroderma renal crisis during pregnancy with very high risks of maternal and fetal morbidity and mortality and the benefits of treatment with ACE inhibitors to both mother and fetus if renal crisis is suspected are highly likely to outweigh the risks of fetotoxicity associated with use [14, 21, 22].

Unfortunately, scleroderma renal crisis can be difficult to distinguish from preeclampsia (abrupt onset hypertension and proteinuria) in the pregnant SSc patient; both conditions carry a high risk of severe maternal and fetal complications if not treated aggressively. In situations where the woman has a history of renal crisis or is at high risk for developing renal crisis (early rapidly progressive renal disease), an immediate trial of ACE inhibitors may be indicated. In cases of profound maternal or fetal distress, emergent delivery may be the most appropriate option followed by initiation of ACE inhibitor therapy. In this situation, the definitive therapy for preeclampsia has been completed (delivery), and ACE inhibitors can be instituted without concern of risk of fetotoxicity of antenatal ACE inhibitor exposure [21]. Renal biopsy may be indicated in cases where the distinction between renal crisis and preeclampsia is necessary for management [23].

## 5. Pulmonary Arterial Hypertension

Pulmonary arterial hypertension (PAH) has been increasingly recognized as a major cause of morbidity and mortality in the SSc population. Estimates of PAH in the SSc population range from 7.85% to 26.7% [24, 25]. In contrast to scleroderma renal crisis, PAH can occur with both limited and diffuse cutaneous disease and may be more prevalent in patients with limited cutaneous disease. PAH in SSc is a result of long standing vasculopathy involving the pulmonary arterial vasculature. Pulmonary arterioles exhibit the same “onion skin” appearance as seen in renal crisis. Symptom onset is often insidious, delaying diagnosis and the introduction of appropriate therapy. Experts recommend routine screening for PAH to ensure early diagnosis and therapy in hopes of preventing remodeling and permanent occlusion of the vessel lumen. 

It is clear that women with PAH are at extremely high risk for severe hemodynamic complications during pregnancy as there is significantly less reserve in the pulmonary arterioles to reduce vascular resistance to accommodate the increased blood volume and cardiac output that occurs during pregnancy. Reports estimate a 36%–50% maternal death rate in women with PAH, with the most vulnerable period occurring with delivery and the first two weeks postpartum [26, 27]. A more recent study suggested a slightly lower maternal death rate of 17%–33%, but this is arguably still unacceptably high [28]. Death is usually due to acute cardiovascular collapse. Rates of preterm delivery with resultant neonatal morbidity and mortality are similarly high in the PAH population. For all of these reasons, women with known PAH should be strongly discouraged from becoming pregnant; additionally, women with SSc (diffuse or limited cutaneous disease) should undergo careful screening for subclinical PAH when considering a future pregnancy. Noninvasive evaluation for PAH includes an echocardiogram looking for a pulmonary arterial pressure of >30 mm Hg at rest or an isolated reduced diffusion capacity (DLCO) in the absence of restrictive lung disease on standard pulmonary function testing [24, 25]. Furthermore, all complaints of dyspnea in pregnant SSc patients should prompt an immediate evaluation for development or worsening of PAH. Assessing diffusion capacity using carbon monoxide is considered safe during pregnancy [29].

If a woman with PAH discovers a pregnancy and wishes to continue with the pregnancy or if PAH is diagnosed during an established pregnancy, careful hemodynamic monitoring and comanagement with pulmonologists experienced with PAH is essential. Case reports have described successful use of epoprostenol and sildenafil during pregnancy [24, 26–28, 30–32]. Anticoagulation with low-molecular-weight heparin is recommended to reduce risk of thromboembolism [26], and some have suggested use of supplemental oxygen to maintain a PO2 greater than 70 mm Hg [26]. Inhaled NO has been used in extreme circumstances during labor and delivery [26, 27]. 

Delivery is a period of extremely high risk in the woman with PAH. Acute hemodynamic changes including increase in cardiac output of 25%–50% during the second stage of labor through delivery with the return of blood volume from the uterus to the main circulation [26, 27]. Increased maternal mortality has been described with general ansthesia [28]. The preferred mode of delivery remains controversial: vaginal delivery is associated with less shifts in blood volume but has a prolonged second stage of labor and issues regarding increased pressure with contractions. Cesarean delivery reduces the second stage of labor and may be necessary in cases of extreme maternal or fetal distress but increases risks of infection and thrombosis [28].

## 6. Raynauds Phenomenon and Digital Ulceration

Of all vascular complications of SSc, Raynauds phenomenon and digital ulcers are most likely to improve during pregnancy. Raynauds phenomenon is characterized by vascular hyperreactivity and vasospasm. However, when digital vasculopathy becomes more fixed, as is often the case in SSc, chronic poor perfusion leads to digital ulceration. Raynauds phenomenon tends to improve during pregnancy, only to worsen postpartum [15]. Less is known about the development of or healing of digital ulcerations. Improvements may be because the increased blood volume and reduced systemic vascular resistance may improve peripheral circulation. Return to pre-pregnancy hemodynamics leads to return of baseline peripheral circulatory complications. A study of pregnancy outcomes among women with primary Raynauds phenomenon (without SSc or other connective tissue diseases) found a slightly increased risk of preterm delivery and smaller weights among full- term infants [33] but concluded that these outcomes did not have any adverse clinical significance for mother or infant. The study did not evaluate the course of Raynaud's phenomenon during the pregnancy or postpartum period.

## 7. Decidual/Placental Vasculopathy

Given the diffuse vasculopathy present in patients with SSc, there are concerns that the same pathophysiologic changes may occur in the placental vasculature. The higher rates of prematurity and small for gestation age babies seen in SSc pregnancies may be a direct result of placental vascular insufficiency. Most studies did not examine placental tissue, thus not allowing for direct correlation between placental vascular abnormalities and adverse pregnancy outcomes. Histopathologic examination of placentas from a limited number of SSc pregnancies has been reported; all of which reported normal placenta weight for gestational age [34–36]. In one study of three placentas from SSc patients (gestational age at delivery between 34 and 38 weeks) found evidence of decidual vasculopathy with stomal fibrosis and infarcts in chronic villi despite an absence of reported adverse pregnancy outcomes [33]. In another case of a pregnancy SSc patient with intrauterine growth restriction was found to have increased resistance in the umbilical artery by dopper examination at 31-week gestational age. Examination of the placenta after delivery at 37 weeks found numerous placental infarcts, placental mesenchymal dysplasia, decreased vascularity, and stromal fibrosis all consistent with decidual vasculopathy [35]. In the largest study to date, 13 placentas from SSc patients were examined and correlated with perinatal outcomes [36]. Five of 13 placentas demonstrated marked decidual vasculopathy, four of which were associated with intrauterine fetal demise between weeks 16 and 30. Chorioamnionitis and accelerated placental maturation complicated the majority of other placentas. These findings are similar to what is seen in pregnancies complicated by pregnancy-induced hypertension. Indeed, in a nationwide study of pregnancy outcomes of SSc patients, 23% carried a diagnosis of pregnancy-induce hypertension [18]. Thus, placental abnormalities may be present in SSc pregnancies, even in the absence of clinical perinatal complications, and these abnormalities may be more severe correlating with perinatal growth restriction and death.

## 8. Conclusion

Healthy pregnancies require extensive pulmonary, cardiovascular, and vascular changes to support the fetus during development. An inability to accommodate such changes due to preexisting pulmonary, cardiac, or vascular disease may lead to pregnancy complications and an inability to support the continuance of the pregnancy. Vasculopathy is a prominent feature of SSc in general and may play an important role in adverse pregnancy outcomes in women with preexisting disease. Women with SSc who are contemplating pregnancy or who discover an inadvertent pregnancy should undergo a thorough evaluation for systemic and organ-specific vasculopathy. Those with a history of scleroderma renal crisis or pulmonary arterial hypertension (or patients at high risk for developing these complications) are at the highest risk for maternal and fetal death and other highly morbid complications of pregnancy. Those with or at high risk for renal or pulmonary arterial vasculopathy should be strongly advised against becoming pregnant and to consider termination if a pregnancy occurs. If a woman wishes to continue with a pregnancy after appropriate counseling of risks, aggressive monitoring and co-management with experts in renal or pulmonary arterial disease is mandatory. Medications such as ACE inhibitors and prostaglandins, which can carry risks for congenital malformations or fetal toxicity, need to be considered as the benefits to both mother and fetus may outweigh known risks of antenatal exposure. Labor and delivery is a very vulnerable period in these cases, and patients may require extended observation in the hospital following delivery to watch for acute cardiovascular collapse in cases of PAH.

However, if after thorough evaluation, a woman with SSc does not appear to have vasculopathy of the internal organs, pregnancy may be considered. Raynaud's phenomenon should not be considered a contraindication to pregnancy in the absence of renal disease or PAH. With careful monitoring by high-risk obstetrics, most women without advanced vasculopathy can expect to have reasonable perinatal outcomes although preterm delivery is common. Advances in neonatology have helped improve the short- and long-term outcomes of premature infants.

Whenever possible, placentas and umbilical cords should be subject to histopathologic examination for evidence of decidual vasculopathy and stromal fibrosis. This will hopefully lead to better understanding of the role of placental vasculopathy in adverse pregnancy outcomes and may additionally provide histopathologic clues to systemic or organ- specific vasculopathy in SSc patients.

## References

[1] Bernatsky S, Joseph L, Pineau CA, Belisle P, Hudson M, Clarke AE (2009). Scleroderma prevalence: demographic variations in a population-based sample. *Arthritis Care and Research*.

[2] Mayes MD, Lacey JV, Beebe-Dimmer J (2003). Prevalence, incidence, survival, and disease characteristics of systemic sclerosis in a large US population. *Arthritis and Rheumatism*.

[3] Carbillon L, Uzan M, Uzan S (2000). Pregnancy, vascular tone, and maternal hemodynamics: a crucial adaptation. *Obstetrical and Gynecological Survey*.

[4] Ganzevoort W, Rep A, Bonsel GJ, De Vries JIP, Wolf H (2004). Plasma volume and blood pressure regulation in hypertensive pregnancy. *Journal of Hypertension*.

[5] Thornburg KL, Jacobson S-L, Giraud GD, Morton MJ (2000). Hemodynamic changes in pregnancy. *Seminars in Perinatology*.

[6] Frederiksen MC (2001). Physiologic changes in pregnancy and their effect on drug disposition. *Seminars in Perinatology*.

[7] Ramsay B, De Belder A, Campbell S, Moncada S, Martin JF (1994). A nitric oxide donor improves uterine artery diastolic blood flow in normal early pregnancy and in women at high risk of pre-eclampsia. *European Journal of Clinical Investigation*.

[8] Karlen JR, Cook WA (1974). Renal scleroderma and pregnancy. *Obstetrics and Gynecology*.

[9] Sood SV, Kohler HG (1970). Maternal death from systemic sclerosis. *Journal of Obstetrics and Gynaecology of the British Commonwealth*.

[10] Younker D, Harrison B (1985). Scleroderma and pregnancy. *British Journal of Anaesthesia*.

[11] Scarpinato L, Mackenzie AH (1985). Pregnancy and progressive systemic sclerosis. Case report and review of the literature. *Cleveland Clinic Quarterly*.

[12] Østensen M (2008). Scleroderma pregnancy: can the price be too high to pay?. *Clinical and Experimental Rheumatology*.

[13] Steen VD, Conte C, Day N, Ramsey-Goldman R, Medsger TA (1989). Pregnancy in women with systemic sclerosis. *Arthritis and Rheumatism*.

[14] Steen VD, Medsger TA (1999). Fertility and pregnancy outcome in women with systemic sclerosis. *Arthritis and Rheumatism*.

[15] Steen VD (2007). Pregnancy in scleroderma. *Rheumatic Disease Clinics of North America*.

[16] Chung L, Flyckt RLR, Colón I, Shah AA, Druzin M, Chakravarty EF (2006). Outcome of pregnancies complicated by systemic sclerosis and mixed connective tissue disease. *Lupus*.

[17] Miniati I, Guiducci S, Mecacci F, Mello G, Matucci-Cerinic M (2008). Pregnancy in systemic sclerosis. *Rheumatology*.

[18] Chakravarty EF, Khanna D, Chung L (2008). Pregnancy outcomes in systemic sclerosis, primary pulmonary hypertension, and sickle cell disease. *Obstetrics and Gynecology*.

[19] Resnik R (2002). Intrauterine growth restriction. *Obstetrics and Gynecology*.

[20] Batal I, Domsic RT, Shafer A (2009). Renal biopsy findings predicting outcome in scleroderma renal crisis. *Human Pathology*.

[21] Cooper WO, Hernandez-Diaz S, Arbogast PG (2006). Major congenital malformations after first-trimester exposure to ACE inhibitors. *The New England Journal of Medicine*.

[22] Easterling TR, Carr DB, Davis C, Diederichs C, Brateng DA, Schmucker B (2000). Low-dose, short-acting, angiotensin-converting enzyme inhibitors as rescue therapy in pregnancy. *Obstetrics and Gynecology*.

[23] Mok CC, Kwan TH, Chow L (2003). Scleroderma renal crisis sine scleroderma during pregnancy. *Scandinavian Journal of Rheumatology*.

[24] Wigley FM, Lima JAC, Mayes M, McLain D, Chapin JL, Ward-Able C (2005). The prevalence of undiagnosed pulmonary arterial hypertension in subjects with connective tissue disease at the secondary health care level of community-based rheumatologists (the UNCOVER study). *Arthritis and Rheumatism*.

[25] Hachulla E, de Groote P, Gressin V (2009). The three-year incidence of pulmonary arterial hypertension associated with systemic sclerosis in a multicenter nationwide longitudinal study in France. *Arthritis and Rheumatism*.

[26] Madden BP (2009). Pulmonary hypertension and pregnancy. *International Journal of Obstetric Anesthesia*.

[27] Huang S, DeSantis ERH (2007). Treatment of pulmonary arterial hypertension in pregnancy. *American Journal of Health-System Pharmacy*.

[28] Bédard E, Dimopoulos K, Gatzoulis MA (2009). Has there been any progress made on pregnancy outcomes among women with pulmonary arterial hypertension?. *European Heart Journal*.

[29] Zavorsky GS, Blood AB, Power GG, Longo LD, Artal R, Vlastos EJ (2010). CO and NO pulmonary diffusing capacity during pregnancy: safety and diagnostic potential. *Respiratory Physiology and Neurobiology*.

[30] Higton AM, Whale C, Musk M, Gabbay E (2009). Pulmonary hypertension in pregnancy: two cases and review of the literature. *Internal Medicine Journal*.

[31] Goland S, Tsai F, Habib M, Janmohamed M, Goodwin TM, Elkayam U (2010). Favorable outcome of pregnancy with an elective use of epoprostenol and sildenafil in women with severe pulmonary hypertension. *Cardiology*.

[32] Leuchte HH, Schwaiblmair M, Baumgartner RA, Neurohr CF, Kolbe T, Behr J (2004). Hemodynamic response to sildenafil, nitric oxide, and iloprost in primary pulmonary hypertension. *Chest*.

[33] Kahl LE, Blair C, Ramsey-Goldman R, Steen VD (1990). Pregnancy outcomes in women with primary Raynaud’s phenomenon. *Arthritis and Rheumatism*.

[34] Ibba-Manneschi L, Manetti M, Milia AF (2010). Severe fibrotic changes and altered expression of angiogenic factors in maternal scleroderma: placental findings. *Annals of the Rheumatic Diseases*.

[35] Papakonstantinou K, Hasiakos D, Kondi-Paphiti A (2007). Clinicopathology of maternal scleroderma. *International Journal of Gynecology and Obstetrics*.

[36] Doss BJ, Jacques SM, Mayes MD, Qureshi F (1998). Maternal scleroderma: placental findings and perinatal outcome. *Human Pathology*.

